# Using the Single Prolonged Stress Model to Examine the Pathophysiology of PTSD

**DOI:** 10.3389/fphar.2017.00615

**Published:** 2017-09-11

**Authors:** Rimenez R. Souza, Lindsey J. Noble, Christa K. McIntyre

**Affiliations:** ^1^Texas Biomedical Device Center, School of Behavioral and Brain Sciences, University of Texas at Dallas, Richardson TX, United States; ^2^Cognition and Neuroscience Program, School of Behavioral and Brain Sciences, University of Texas at Dallas, Richardson TX, United States

**Keywords:** animal models, fear, glucocorticoids, memory, PTSD, SPS, stress, extinction

## Abstract

The endurance of memories of emotionally arousing events serves the adaptive role of minimizing future exposure to danger and reinforcing rewarding behaviors. However, following a traumatic event, a subset of individuals suffers from persistent pathological symptoms such as those seen in posttraumatic stress disorder (PTSD). Despite the availability of pharmacological treatments and evidence-based cognitive behavioral therapy, a considerable number of PTSD patients do not respond to the treatment, or show partial remission and relapse of the symptoms. In controlled laboratory studies, PTSD patients show deficient ability to extinguish conditioned fear. Failure to extinguish learned fear could be responsible for the persistence of PTSD symptoms such as elevated anxiety, arousal, and avoidance. It may also explain the high non-response and dropout rates seen during treatment. Animal models are useful for understanding the pathophysiology of the disorder and the development of new treatments. This review examines studies in a rodent model of PTSD with the goal of identifying behavioral and physiological factors that predispose individuals to PTSD symptoms. Single prolonged stress (SPS) is a frequently used rat model of PTSD that involves exposure to several successive stressors. SPS rats show PTSD-like symptoms, including impaired extinction of conditioned fear. Since its development by the Liberzon lab in 1997, the SPS model has been referred to by more than 200 published papers. Here we consider the findings of these studies and unresolved questions that may be investigated using the model.

## Introduction

The focus of this *Frontiers in Pharmacology* Research Topic is the neural mechanisms of memory. Memory is a fundamental process in all animals, as it allows survival and success through learned adaptive behaviors. However, some highly stressful experiences can lead to maladaptive fear, anxiety, and protracted periods of suffering like in Posttraumatic Stress Disorder (PTSD). A hallmark symptom of this condition is re-experiencing the traumatic event, suggesting that the problem lies in the mechanisms controlling storage and expression of the traumatic memories. In this mini-review, we will discuss prospective research studies performed in animals to uncover clues about how traumatic experiences can lead to the pathophysiology of PTSD. We also outline some current limitations, knowledge gaps, and areas that require further investigation.

## Single-Prolonged Stress

**FIGURE 1:**
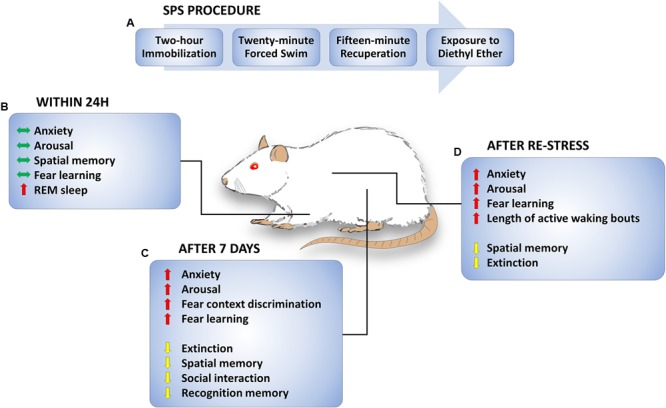
Single prolonged stress (SPS) procedure and SPS-induced behavioral changes. **(A)** Timeline of SPS procedure. On a single day, rats are subjected to a 2-h immobilization followed immediately by a 20-min forced swim. Rats are given a brief period of recuperation and then subjected to diethyl ether until they are anesthetized and unresponsive. **(B)** Behavioral changes observed up to 1 day later. Anxiety, arousal, spatial memory, and fear learning are unchanged. Acute increase in REM sleep and transition to REM sleep is observed. **(C)** Behavioral changes 1 week later. Anxiety, arousal, fear context discrimination, and fear learning are increased. On the other hand, extinction, spatial memory, social interaction, and recognition memory are decreased a week after SPS. **(D)** Behavioral changes following re-stress. Enhanced anxiety, arousal, fear learning, and sleep disturbances remain observed following re-stress, while extinction and spatial memory are impaired. Green, red, and yellow arrows indicate no “changes observed”, “increase”, and “decrease”, respectively.

Single prolonged stress (SPS) is a frequently used rat model of PTSD. Since its initial description 20 years ago (Liberzon et al., 1997), the SPS procedure has been referred to by over 200 peer reviewed studies. Although it is called a “single” prolonged stress, the procedure is comprised of successive, multi-modal stressors (**Figure 1**). The prolonged stress begins with a 2-h immobilization period that is immediately followed by a forced-swim experience, lasting 20 min, and then a brief loss of consciousness induced by ether exposure. After recovery, rats remain undisturbed for 7 days (Liberzon et al., 1997). In some cases, they are socially isolated (individually housed) during this period (Knox et al., 2012a). When rats undergo auditory or contextual fear conditioning 7 days after this procedure, they demonstrate impaired retention of extinction learning and the conditioned fear response persists longer than it does with fear conditioning alone (Knox et al., 2012a). This approach can be useful for modeling PTSD-like symptoms because those who experience multiple traumas, or a trauma early in life, are more susceptible to developing PTSD following a later traumatic event (Maercker et al., 2004; Anda et al., 2006; Kilpatrick et al., 2013).

Precisely how a previous trauma predisposes individuals to the development of PTSD remains unknown. The first trauma or traumas may simply make an individual more anxious, in general, or more sensitive to future stressors. Alternatively, a previous stressor may set the brain up to acquire, store, or retrieve traumatic memories differently, going forward. Some researchers have hypothesized that an impairment in the recall of fear extinction learning may be an underlying cause of PTSD symptoms (Milad et al., 2008, 2009). The SPS rat model provides an opportunity for testing these hypotheses.

## Effects of SPS On Behavior and the Brain

Many findings suggest that SPS produces behavioral and physiological symptoms that are similar to those observed in PTSD (Liberzon et al., 1997; Kohda et al., 2007; Yamamoto et al., 2010; Knox et al., 2016). Examples of behavioral effects of SPS are illustrated in **Figure 1**. SPS rats demonstrate sleep abnormalities (Vanderheyden et al., 2015) enhanced anxiety (Han et al., 2014; Liu et al., 2016), arousal (Khan and Liberzon, 2004), and fear learning (Iwamoto et al., 2007; Keller et al., 2015b) as well as impaired spatial and recognition memory, social interaction (Kohda et al., 2007; Wen et al., 2016) and fear extinction (Knox et al., 2012a; Keller et al., 2015b). Most changes are observed 7 days, but not 1 day, after exposure to the SPS procedure, suggesting that behavioral and cellular changes promoted by SPS are time-dependent (Liberzon et al., 1999a; Knox et al., 2016; Wu et al., 2016). Although it has been demonstrated that partial SPS does not generate extinction impairments (Knox et al., 2012b), the critical features of the SPS procedure for development of a PTSD-like phenotype remain unclear. For example, the passage of time alone may be sufficient for an incubation or sensitization effect following the SPS procedure, or a second stressful experience may be necessary to produce cumulative effects on behavior. In **Figure 1**, behavioral effects of SPS are categorized by the time of testing, i.e., whether testing occurred after SPS, SPS + 7 days (with or without social isolation), or SPS + 7 days + an additional stressor. Though there are variations in some SPS procedures (i.e., social isolation vs. group housing), many studies report consistent SPS effects. For example, social isolation during the quiescent period (Harada et al., 2008) and group housing during the quiescent period (Imanaka et al., 2006) both produced an enhancement in contextual fear conditioning following SPS.

### Impaired Extinction of Conditioned Fear

One explanation for the persistence of fear, anxiety, avoidance, and re-experiencing symptoms in PTSD is that some individuals have strong traumatic memories that are less susceptible to extinction. Indeed, some studies of PTSD patients show enhanced conditioned fear (Blechert et al., 2007; Glover et al., 2011; Norrholm et al., 2011), and several animal studies demonstrate an enhancement in contextual fear conditioning following SPS (Iwamoto et al., 2007; Kohda et al., 2007; Keller et al., 2015b). However, others have reported extinction impairments despite normal acquisition of conditioned fear (Milad et al., 2008, 2009; Eskandarian et al., 2013; Vanderheyden et al., 2015; Knox et al., 2016). Using skin conductance responses as a measure of conditioned fear, Milad et al. (2008, 2009) found that PTSD patients showed normal fear conditioning and within-session extinction, but poor retention of extinction on later tests. In rats, prior exposure to the SPS procedure impaired extinction of both cued (Knox et al., 2012a; George et al., 2015; Keller et al., 2015b) and contextual fear conditioning (Yamamoto et al., 2008; Knox et al., 2012a; Matsumoto et al., 2013), whereas acquisition of conditioned fear and extinction within a session were not affected (Knox et al., 2012a,b). Given the evidence that within-session conditioning and retrieval are normal, these findings suggest that consolidation of the extinction memory is impaired in human PTSD patients and in SPS rats. Neurobiological changes that could contribute to impairments in behavior and fear extinction are discussed below (**Table 1**).

**Table 1:** Cellular changes in three key areas controlling memory and emotionality after single prolonged stress (SPS) model of PTSD.

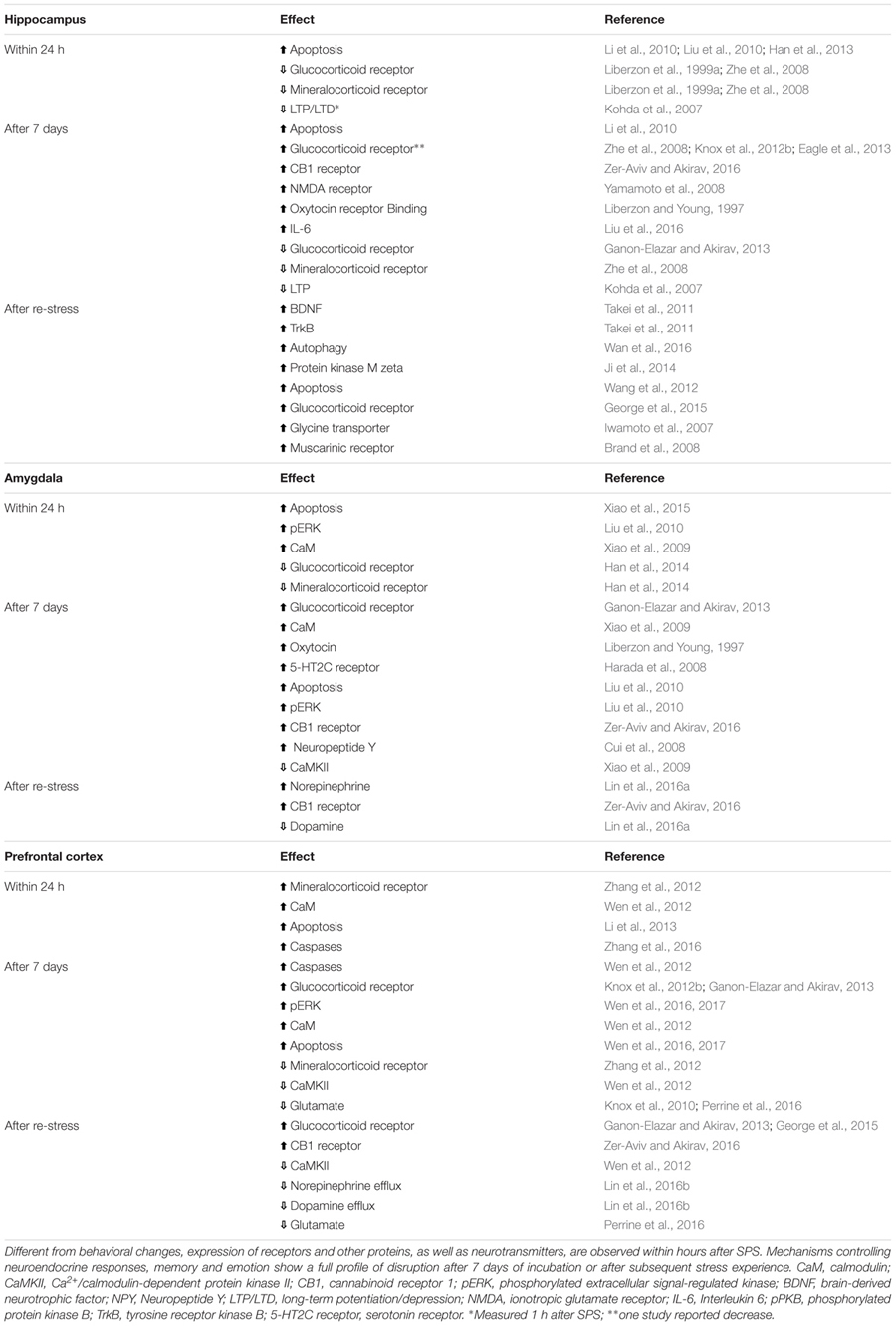

### Hippocampus

The hippocampus plays a role in storing fear memories and in mediating stress responses (Phillips and LeDoux, 1992; McEwen, 2007). Not surprisingly, the hippocampus is highly sensitive to chronic stress (McEwen, 2007). This is confirmed by functional magnetic resonance imaging (fMRI) studies demonstrating that PTSD patients have a smaller hippocampal volume than healthy controls (Bremner et al., 1995; Stein et al., 1997), although some research suggests that a lower hippocampal volume may represent a risk factor for PTSD (Gilbertson et al., 2002). These findings indicate that reduced hippocampal function might be associated with resistant memory impairments in PTSD.

To our knowledge, no studies have examined the effect of the SPS procedure on hippocampal volume, however, the hippocampus has been the subject of many investigations. Enhanced apoptosis, a phenomenon involved in programed cell death that results in morphological changes, is observed in the hippocampus shortly after SPS, and persists after the undisturbed phase, and after a subsequent stressor (Li et al., 2010; Liu et al., 2010; Wang et al., 2012; Han et al., 2013). Re-stress after SPS also enhances autophagosomes and autophagy-related markers (Wan et al., 2016). Likewise, studies using the SPS model show evidence of enhanced oxidative stress and inflammation (Schiavone et al., 2013). For example, IL-6, malondialdehyde, NOX2, and 4-hydroxynonenal contribute to apoptotic cell death in the hippocampus following SPS (Li et al., 2010; Wang et al., 2012; Han et al., 2013; Liu et al., 2016). Balance and expression of GR and MR receptors is disrupted in the hippocampus of SPS rats. Thus, while decreased expression of GR and MR is observed shortly after SPS (Liberzon et al., 1999a; Zhe et al., 2008), increased expression of these receptors is observed after a week or after re-stress (Zhe et al., 2008; Knox et al., 2012b; Eagle et al., 2013; George et al., 2015). Synaptic plasticity-related mechanisms are also influenced by SPS. Both LTP and LTD are decreased after SPS (Kohda et al., 2007), while NMDA receptor expression is enhanced (Yamamoto et al., 2008). In a recent study using c-Fos expression, Knox et al. (2016) found that SPS disrupted the inhibition of ventral hippocampal activity during extinction retrieval as well as the functional connectivity within the dorsal hippocampus during extinction learning.

### Amygdala

The amygdala is also involved in the control of fearful states and learning of emotional experiences. Imaging studies have revealed that PTSD patients show exaggerated amygdala activity in response to trauma-related cues or unrelated arousing stimuli and during new fear learning (Liberzon et al., 1999b; Dunsmoor et al., 2011; Sartory et al., 2013), supporting the notion that enhanced amygdala activity could be involved in impaired extinction learning or generalization of fear responses.

Studies using the SPS model demonstrate changes in the amygdala starting a day after the procedure (**Table 1**). Increased apoptosis and downstream signals, like phosphorylated extracellular signal–regulated kinases, glucose-regulated protein 78 (GRP78) and caspases 3, 9, and 12 expression, were observed in the amygdala 1 day after SPS, and some reached peak levels 7 days later (Liu et al., 2010; Xiao et al., 2011, 2015), suggesting that SPS-induced morphological and connectivity changes may precede the behavioral and memory deficits observed after the 7-day period. Potentiated-fear learning following SPS was paralleled by an early decrease in GR and MR receptors in the amygdala, as well as by blunted LTP and decreased colocalization of GR and MR receptors 1 week later (Kohda et al., 2007; Han et al., 2014). Intracellular calcium levels are changed shortly after SPS and the effect persists for 1 week (Xiao et al., 2009). Acute changes in calmodulin (CaM) and calcium-CaM kinase II (CaMKII), two messengers involved in Ca^2+^ homeostasis and signaling processes related to learning and memory, were up- and downregulated, respectively, within 1 day of SPS (Xiao et al., 2009), indicating that SPS disrupts fundamental mechanisms of cell signaling, which may lead to amygdala hyperactivity, enhanced fear expression and impaired extinction of conditioned fear.

### Prefrontal Cortex

Inhibition of amygdala hyperactivity and cognitive flexibility are important prefrontal cortex functions that are implicated in PTSD susceptibility and symptoms (Kitayama et al., 2006; Shin et al., 2006; Gold et al., 2011). This notion is supported by functional imaging studies showing a reduced activity of the medial prefrontal cortex and anterior cingulate cortex in PTSD patients during presentation of trauma-related and non-related aversive stimuli (Shin et al., 2006; Etkin and Wager, 2007; Gold et al., 2011). Moreover, the volume of the ventromedial prefrontal cortex and the anterior cingulate cortex is reduced in individuals with PTSD (Kitayama et al., 2006; Kasai et al., 2008; Karl and Werner, 2010). Abnormal morphological changes in the pathway from the anterior cingulate cortex to the amygdala was also found in PTSD patients (Kim et al., 2006), suggesting that a series of changes in the normal control of the fearful states or behavioral flexibility by the frontal cortex may be involved in the pathophysiology of PTSD.

Evidence for similar changes in the prefrontal cortex of rats submitted to the SPS model remains sparse. As in the hippocampus and amygdala, neuronal apoptosis and dysregulation of autophagic activity in the prefrontal cortex appears 1 day after SPS (Li et al., 2013; Wen et al., 2016; Zheng et al., 2017). Elevated levels of protein kinase RNA-like endoplasmic reticulum kinase (PERK), activating transcription factor 6 (ATF6), inositol-requiring enzyme 1 (IRE1) in the endoplasmic reticulum (ER), glucose-regulated protein (GRP) 94 and apoptosis-related caspase-12 are involved in the persistent apoptotic profile seen 1 week after SPS (Li et al., 2013; Zhao et al., 2014; Wen et al., 2016, 2017). Unbalanced control of calcium indicates that intracellular messengers controlling neuronal excitability are disrupted following SPS (Wen et al., 2012). This is corroborated by studies showing decreased levels of glutamate in the prefrontal cortex 1 week after SPS or re-stress (Knox et al., 2010; Perrine et al., 2016). The concentration of MRs is elevated 1 day after SPS (Zhang et al., 2012), while GR expression is enhanced 1 week later and after re-stress (Knox et al., 2012b; Ganon-Elazar and Akirav, 2013; George et al., 2015), indicating temporally distinct disturbances in stress-related systems.

Decreased volume and integrity of prefrontal sub-regions have been reported in PTSD patients (Rauch et al., 2003; Woodward et al., 2006). Similarly, SPS disrupts normal activity of the infralimbic region of the medial prefrontal cortex before re-stress (Knox et al., 2016), suggesting that SPS could predispose the prefrontal cortex to dysfunctional activity during fear learning and/or subsequent extinction trials. However, since different regions of the prefrontal cortex control distinct aspects of fear learning and extinction, additional studies are needed for a better understanding about changes that can be predisposing factors or consequences of the trauma.

### Effects of SPS on HPA-Axis

Early research on the pathophysiology of PTSD identified a decrease in cortisol levels (Yehuda et al., 1990). Later studies demonstrated that administration of low doses of dexamethasone produced suppression of plasma cortisol, indicating that the hypothalamus-pituitary-adrenal cortex (HPA) axis may become sensitive to negative feedback in PTSD patients (Yehuda et al., 1995). Similarly, enhanced suppression of the HPA-axis is observed in rats 7 days after SPS (Liberzon et al., 1997, 1999a). The data currently available suggest that the enhanced glucocorticoid negative feedback observed in SPS may be linked to overexpression of GR and a reduced expression of MR in key areas mediating activity of the HPA-axis during stress (Liberzon et al., 1999a; Zhe et al., 2008; Eagle et al., 2013).

Changes in the HPA-axis may contribute to PTSD symptoms by interfering with extinction of conditioned fear. For example, exogenous administration of stress-levels of cortisol can impair the retrieval of long-term memories (de Quervain et al., 1998), but the same treatment enhances consolidation of new memories (McGaugh and Roozendaal, 2002; de Quervain et al., 2009). These findings suggest that SPS-induced enhanced suppression of the HPA-axis may have the opposite effect, perpetuating the fear memory by facilitating retrieval of the traumatic memory and impairing consolidation of extinction memory (de Quervain et al., 2017). However, a few studies have dissociated GR upregulation and extinction impairments in the SPS model. A significant increase in GR expression was observed in the hippocampus and prefrontal cortex 7 days after partial SPS (e.g., forced swimming and ether exposure) that did not impair extinction of conditioned fear (Knox et al., 2012b). These results indicate that glucocorticoid receptor expression must reach a threshold in order to interfere with the consolidation of extinction, or there is another SPS-related change that influences the extinction of conditioned fear. Consistent with the view that enhanced suppression of the HPA-axis and the resulting decrease in circulating glucocorticoids predisposes animals to the PTSD phenotype, Keller et al. (2015b) found that inhibition of corticosterone synthesis prior to fear conditioning exacerbated the extinction impairment in SPS rats.

Taken together, these findings indicate that the SPS model is a useful tool for studying the role of the HPA-axis in PTSD. Future studies should examine the full extent of HPA-axis changes, including the evaluation of SPS effects on circulating glucocorticoid levels. Further studies may be designed to determine whether HPA-axis dysfunction is a predisposing factor or a consequence of traumatic experience.

## Limitations

Many PTSD-like effects have been identified in rats exposed to SPS. However, seemingly subtle deviations in the procedure may have significant consequences on behavior and physiology (Knox et al., 2012b). In this review, we have sorted the behavioral and physiological consequences of SPS by the time of testing. Some effects are transient, and some emerge after 7 days or a re-stress experience, suggesting that the effects of SPS are time- and experience-dependent. Variations on SPS parameters can be utilized to identify factors producing maladaptive fear and arousal states. Future studies are needed to determine the relative contributions of the passage of time and stress experience to these SPS-related changes.

Here, we also describe evidence that extinction impairments are a common feature of PTSD and the rat SPS model of PTSD. A major caveat is that human females are two times more likely to develop PTSD following a traumatic event (Kessler et al., 2005), yet SPS-induced deficits in extinction are only seen in male rats. In one study that investigated sex differences in the SPS model, Keller et al. (2015a) demonstrated that SPS affects GR expression in the dorsal hippocampus in females, but extinction retention deficits were observed only in males, suggesting that female rats are more resilient to the memory extinction effects of SPS. Such differences may be indicative of a sexually divergent response to conditioned fear. Emerging evidence indicates that female rats express fear by darting rather than freezing (Gruene et al., 2015), indicating that reliance on freezing as a single measure of fear may be misleading.

## Conclusion

Although we have focused on factors contributing to extinction impairments, the SPS model can be used to investigate hypotheses about the biological causes of other debilitating symptoms such as social withdrawal, heightened anxiety, elevated startle response, hypervigilance, and sleep disturbances. Though the SPS model is a useful tool to study the PTSD symptomatology, additional studies are needed to examine sex differences, the timing of onset and persistence of symptoms, as well as the features of the SPS procedure that are necessary for the development of PTSD-like symptoms. Given the understanding that all models have limitations, it is encouraging to note that several other animal models demonstrate extinction impairments and PTSD-like symptoms (Izquierdo et al., 2006; Matsumoto et al., 2008; Wilber et al., 2009; Goswami et al., 2010; Long and Fanselow, 2012). Utilization of multiple animal models of PTSD and meticulous examination of PTSD-like symptoms will be critical to unfold the pathophysiology of PTSD, and lead to novel and efficient therapeutic strategies.

## Author Contributions

All authors have been studying the Single Prolonged Stress (SPS) rat model of PTSD in the lab for over 1 year. LN and CM met to discuss writing a mini review on the subject of SPS effects on the brain that may be responsible for the PTSD-like symptoms that we and others have observed in this model. We came up with an outline and invited RS to contribute a summary of physiological effects of SPS. LN wrote about behavioral effects. CM combined both portions, edited, and added some discussion. RS produced the figure and table.

## Conflict of Interest Statement

The authors declare that the research was conducted in the absence of any commercial or financial relationships that could be construed as a potential conflict of interest.
